# *In Vitro* Screening of Medicinal Plants Used in Mexico as Antidiabetics with Glucosidase and Lipase Inhibitory Activities

**DOI:** 10.1155/2012/701261

**Published:** 2012-10-02

**Authors:** Guillermo Ramírez, Miguel Zavala, Julia Pérez, Alejandro Zamilpa

**Affiliations:** ^1^Biological and Health Sciences Ph.D. Program, Division of Biological Sciences and Health, Universidad Autónoma Metropolitana-Xochimilco, 14387 Mexico, DF, Mexico; ^2^Southern Biomedical Research Center (IMSS), Argentina 1, Col. Centro, 62790 Xochitepec, MOR, Mexico; ^3^Department of Biological Systems, UAM-Xochimilco, Calzada del Hueso 1100, Col. Villa Quietud, 04960 Mexico, DF, Mexico

## Abstract

This work shows the inhibitory effect on glucosidase and lipase enzymes of 23 medicinal plants described as traditional treatments for diabetes in several Mexican sources. Hydroalcoholic extracts of selected plants were evaluated at 1 mg/mL for glucosidase and 0.25 mg/mL for lipase inhibitory activities, respectively. *Camellia sinensis*, acarbose, and orlistat were used as positive controls. Dose-response curves were done with the most active species. Sixty percent of all tested extracts inhibited more than 25% of **α**-glucosidase activity. *C. sinensis* displayed an inhibition of 85% (IC_50_ = 299 **μ**g/mL), while *Ludwigia octovalvis* and *Iostephane heterophylla* showed the highest inhibition (82.7 %, IC_50_ = 202 **μ**g/mL and 60.6%, CI_50_ = 509 **μ**g/mL, resp.). With respect to lipase activity, *L. octovalvis* and *Tecoma stans* were the most inhibiting treatments (31.4%, IC_50_ = 288 **μ**g/mL; 27.2%, IC_50_ = 320 **μ**g/mL), while *C. sinensis* displayed 45% inhibition (IC_50_ = 310 **μ**g/mL). These results indicate that a high proportion of plants used in Mexico as treatment for diabetes displays significant inhibition of these digestive enzymes.

## 1. Introduction

Since the second half of the 20th century, most societies are experiencing an epidemic-like increase in chronic degenerative diseases, mainly cardiovascular, cancer, and type 2 diabetes mellitus. In 2008, type 2 diabetes mellitus (t2DM) world cases were estimated at 348 million individuals, with a striking increase among children and adolescents living in low- and middle-income countries [1]. Such global surveys show that in spite of many important discoveries about the pathophysiology of the disease, reported in the last 15 years, its incidence continues to climb and surpasses even the boldest projections.

Several new types of drugs have been introduced as treatments in the last decade (dipeptidyl peptidase-4 inhibitors, glucagon-like peptide-1 analogs, and cannabinoid receptor type 1 antagonists) and it seems that glucose renal reabsorption inhibitors will be the next available pharmacological therapy [2]. While bariatric surgery is the most successful approach in some cases, this measure is invasive, somewhat risky (0.5–3% mortality), and very expensive [3]. Glucosidase and lipase inhibitors have been available for a long time as prescription medicines, but their use is infrequent as a treatment for diabetes. The leading glucosidase inhibitor, acarbose, is used scarcely due to its low efficacy in decreasing glycemic levels; the lipase inhibitor orlistat is approved for weight loss but is not indicated as a diabetes control measure. Both compounds elicit unpleasant side effects and are not well accepted by both patients and physicians [4]. On the other hand, it has been shown that some plant preparations, containing glucosidase and/or lipase inhibitors, are devoid of these side effects but are still clinically useful (touchi, green tea) [5, 6]. Our interest in glucosidase inhibitors led us to search for lipase-inhibiting species, following the glucolipotoxicity hypothesis, which states that chronic or postprandial glucose and free fatty acids, in supraphysiological blood concentrations, contribute to beta cell failure [7]. This work explores whether a sample of medicinal plant extracts, selected by the Mexican ethnomedical knowledge as “antidiabetic,” contain significant levels of both inhibiting enzymatic activities and if these *in vitro *assays are able to identify novel candidates for further phytochemical and *in vivo* pharmacological analyses aimed at developing prototypes of phytodrugs for diabetes control.

## 2. Materials and Methods

### 2.1. General

Corn starch (S-4186); 5,5′-dithiobis(2-nitrobenzoic acid) (DTNB, Ellman's reagent; D-8130); pancreatic lipase (type II, crude, from porcine pancreas, L-3126); 2,3-dimercapto-1-propanol tributyrate (DMPTB, 97%; cat : 282413); albumin (fatty acid free, bovine, A-6003) were obtained from Sigma-Aldrich (St. Louis, MO, USA). Acarbose (Sincrosa, Alpharma, Mexico City, Mexico) and orlistat (Lysthin, PsicoFarma, Mexico City, Mexico) were used as positive controls. Glucose was measured with a quantitation kit (Glucosa-TR) from Spinreact (Girona, Spain). Solvents were obtained from Merck (Darmstadt, Germany); miscellaneous chemicals (salts, buffers, solvents) were purchased either from Merck or Sigma.

### 2.2. Bibliographical Sources Used for Plant Selection

The ethnomedical compilations of Cano [8], Aguilar and Xolalpa [9], Andrade-Cetto and Heinrich [10], and Romero-Cerecero et al. [11] were consulted to select species used as treatment for diabetes in Mexico. *L. octovalvis* was incorporated in view of information from healers and plant dealers from the south of the state of Morelos, México (Xochitepec, Jojutla, and Zacatepec areas).

Most of the 23 vegetal species were collected in the state of Morelos, Mexico, and voucher specimens were deposited at the IMSS Herbarium (IMSSH), where identity was assessed by Professor Abigail Aguilar, curator. *Annona squamosa* L. was identified *in situ* by Juan Carlos Juarez Delgado from HUMO Herbarium, Autonomous University of Morelos (UAEM). *Camellia sinensis* (Yamamotoyama, Pomona, USA) was bought at a Japanese specialty store and used as a vegetal positive control (*Cs*HAE). 

### 2.3. Preparation of Hydroalcoholic Extracts

Vegetal materials (aerial parts, leaves, or seeds) were dried in a dark room at 25–30°C for 15 days. The dry material was ground to obtain 4–6 mm particles (diameter). Samples of 25 g of all these plants (20 g for *Hintonia latiflora*) were exhaustively extracted by maceration with 250 mL of an ethanol solution (60%) at 50°C for 2 hours (three times). After filtration, the solvent was removed under reduced pressure distillation. Semisolid extracts were finally lyophilized and stored at 4°C in air-tight centrifuge tubes until needed. Most species produced yield over 20% (Table 1).

### 2.4. Glucosidase Inhibition

Glucosidase inhibition assays were performed in quadruplicate as previously reported [12], at an extract concentration of 1 mg/mL. In brief, cornstarch (12.5 mg/mL) was digested by crude enzyme (homogenate from Sprague Dawley rats' intestinal mucosa) at 37°C for 10 minutes and released glucose was quantified by a glucose oxidase-based clinical reagent (SPINREACT), following manufacturer's directions. All inhibitions were calculated as percentage of uninhibited control reactions.

### 2.5. Lipase Inhibition

Lipase inhibition assay was adapted from the method reported by Choi et al. [13]. The assay is based on the spectrophotometric quantification of free thiols with chromogenic 5, 5′-dithiobis(2-nitrobenzoic acid) (DTNB, Ellman's reagent), released by porcine pancreatic lipase from the 2,3-dimercapto-1-propanol tributyrate substrate (DMPTB, 97%).

The reaction mixture contained 0.2 mM DMPTB in 50 mM TRIS-HCl, pH 7.2, 2 mM CaCl_2_, 0.1 M NaCl, 0.06% Triton X-100, and 0.8 mM DTNB (in DMSO).

The porcine lipase was prepared as a stock at 10 mg/mL in TRIS-HCl 20 mM, pH 6.2, bovine serum albumin (BSA; 1 mg/mL) and 0.1 M NaCl, and stored at −80°C. The working samples of enzyme were diluted to 2 mg/mL in BSA (1 mg/mL), kept at 4°C, and were used during 4-5 hours. The assays were performed at 37°C and were started by adding 10 microliters of enzyme solution to 790 microliters of reaction mix, containing 0.25 mg/mL of plant extract in 50% DMSO or controls. The absorbance changes were recorded for up to 6 minutes at 412 nm, plotted in Excel (Microsoft), and the initial slope was employed as the velocity of the reaction.

### 2.6. HPLC Analysis

Solutions (3 mg/mL) of the most active extracts were analyzed using a HPLC system (Waters Co., Milford, MA, USA) with a photodiode array detector (Waters 2996). Separation was carried out using a RP-18 Supersphere (Merck) column (120 mm × 4 mm; 5 *μ*m) with the following solvent ratios for the mobile phase, where solvent A is water and solvent B corresponds to acetonitrile: A : B = 100 : 0 (0–3 min); 90 : 10 (4-5 min); 80 : 20 (6–9); 0 : 100 (10–13 min); 100 : 0 (14-15 min). The flow rate was 1 mL/min and detection wavelength was scanned at 190–600 nm. The major compounds were analyzed according to their UV spectra and retention time. 

## 3. Results

The Ethnobotanical Veracruz Atlas [8] listed 49 species as “antidiabetic” while Aguilar and Xolalpa [9] and Andrade-Cetto and Heinrich [10] reported 178 and 306 species, respectively. Romero-Cerecero [11] reports 64 species used in the state of Morelos. *Ludwigia octovalvis* (“clavillo”) was found to be mentioned as a species of emerging local use by some diabetic patients and plant dealers. Species mentioned in at least two sources were selected, acquired, and processed to obtain their hydroalcoholic extract.

The test concentration of the extracts was adjusted to obtain a 0–85% range of glucosidase inhibition. As shown in Table 1, the vegetal positive control using *C. sinensis* hydroalcoholic extract (*Cs*HAE) inhibited *α*-glucosidase activity significantly (IC_50_ = 299 *μ*g/mL). Acarbose displayed >95% inhibition at 10 *μ*g/mL.

**Figure 1:**
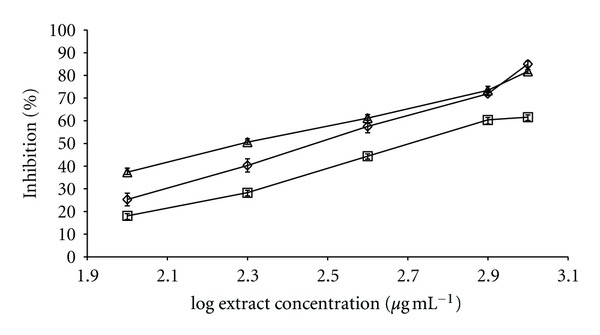
Dose-response curves of the hydroalcoholic extracts from *Camellia sinensis* (◯), *Ludwigia octovalvis* (*▵*), and *Iostephane heterophylla *(□) leaves in the *in vitro α*-glucosidase inhibition model. *x*-axis = log concentration in *μ*g·mL^−1^; *y*-axis = inhibition percentage.

We chose an initial concentration of 1 mg/mL^−1^ for the screening stage. The most active species (inhibition > 50%, Table 1) were further tested at a lower concentration (0.5 mg/mL^−1^, Table 2) and the two species with the highest inhibitory activity (*L. octovalvis* and *Iostephane heterophylla *Benth) were compared with the control (*Camellia sinensis*) in a dose-response curve (Figure 1). *L. octovalvis* showed the lowest IC_50_ (202 *μ*g mL^−1^), followed by *C. sinensis* (299 *μ*g mL^−1^) and *I. heterophylla* (509 *μ*g mL^−1^), respectively (Table 2). 

**Figure 2:**
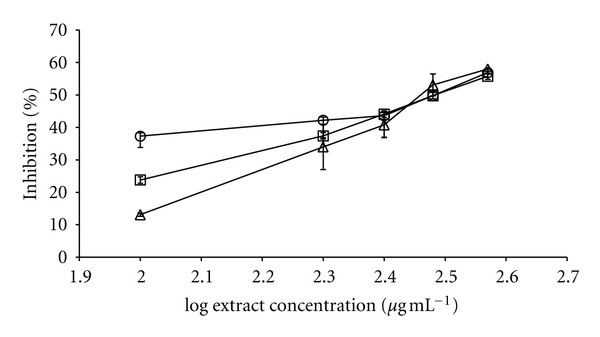
Dose-response curves of the hydroalcoholic extracts from *Camellia sinensis* (◯), *Ludwigia octovalvis* (*▵*), and *Tecoma stans *(□) leaves in the *in vitro* lipase inhibition model. *x*-axis = log concentration in *μ*g·mL^−1^; *y*-axis= inhibition percentage.

Then we examined the effects of all selected extracts on lipase inhibition activity evaluated at 0.25 mg/mL. In this case, the pharmaceutical drug orlistat and *Cs*HAE were used as reference. An initial concentration of 0.25 mg/mL was chosen for all plant-screening purposes, since higher concentrations of some extracts strongly absorbed at the employed wavelength. Under these conditions, the reference extract (*C. sinensis*) displayed 45.6% inhibition of this digestive enzyme. The most active species were compared with the control (*Camellia sinensis*) in a dose-response curve (Figure 2). *C. sinensis*, *L. octovalvis*, and *Tecoma stans* (L.) H.B. & K. gave similar IC_50_ values (310, 288, and 320 *μ*g/mL). In these conditions, orlistat gave an IC_50_ = 142 *n*g/mL. 

**Figure 3:**
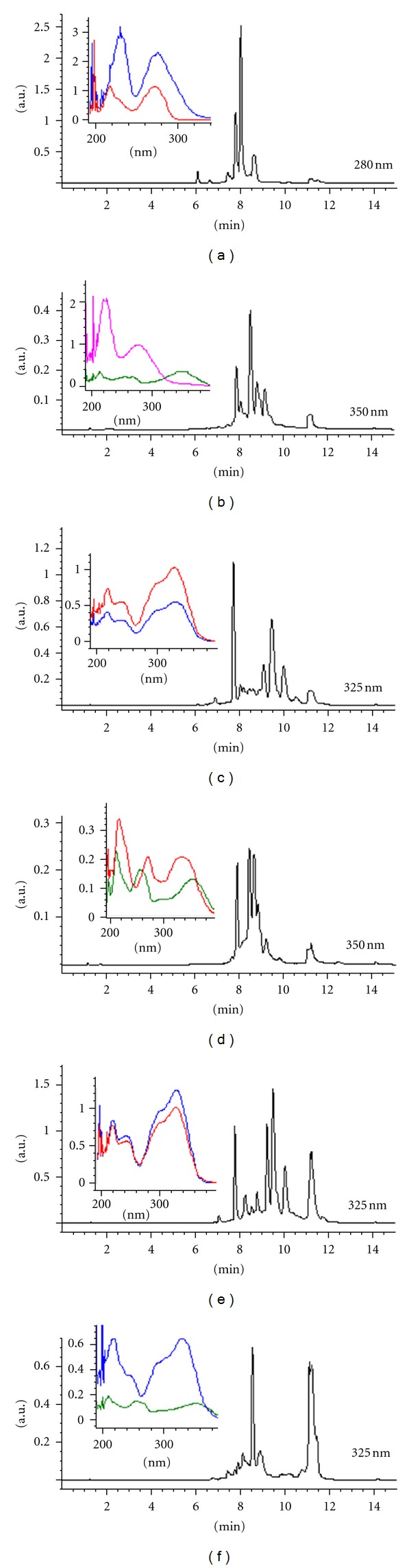
HPLC chromatograms of the reference plant and the most active species—(a) *C. sinensis*, (b) *L. octovalvis*, (c) *I. heterophylla*, (d) *A. farnesiana*, (e) *A. absinthium*, and (f) *T. stans. *

The extracts of the reference plant and the most active species: *Camellia sinensis*,* Ludwigia octovalvis*,* Iostephane heterophylla*,* Acacia. farnesiana*, *Artemisia absinthium*, and *Tecoma stans* were analyzed by DAD-HPLC. The obtained spectra were interpreted by the presence of *λ* max 220, 255, 275, 325, 340, and/or 360 nm. The profile of *C. sinensis* shows *λ* max = 220 and 275 nm, associated with catechins, compounds widely reported in this species [14]. Interestingly, *L. octovalvis* has peaks with similar UV absorption spectrum (*λ* max = 220 and 275 nm), and also others characteristic of flavonols (*λ* max = 210, 255, and 355 nm; Figure 3). Both species show very high inhibition activities. The *A. farnesiana *and* T. stans *UV spectrum profiles show flavonol and flavone patterns (*λ* max = 210, 255, 350 nm and 210, 275, 330 nm resp.), while *A. absinthium*, *C. ternifolia*, and *I. heterophylla *havea profile with peaks associated with caffeoyl derivatives (*λ*max⁡ = 210 and 325 nm) [15, 16].

## 4. Discussion

The scientific literature contains many reports of the *in vivo* antidiabetic activities (mostly hypoglycemic or antihyperglycemic) of medicinal plants. Nevertheless, only few of them describe some action mechanism present in the extract. This survey was designed to search for the inhibition of two enzyme activities in a sample of ethnomedically selected species, considering that they may be part of their antidiabetic properties. This approach is aimed at mildly reducing carbohydrate and lipid digestion and absorption, thus decreasing the hyperglycemia and hyperlipemia peaks. Both measures have been shown to be useful in decreasing the risk of developing diabetes [17, 18]. 

Besides previously reported species, we obtained information from sources in the southern area of the state of Morelos, on the use of *Ludwigia octovalvis* as an antidiabetic. This phenomenon may reflect the empirical search of the population to find additional therapeutic resources for diabetes control.

Two species were found with the highest levels of inhibition in both activities: *Artemisia absinthium* (ajenjo) and *L. octovalvis *(clavillo). The first plant has many traditional uses: It is reported as an antiparasitic, antimicrobial, and hepato- and neuroprotective. While this plant has been reported as rich in flavonols (kaempferol and quercetagetin derivatives) and essential oils like thujone, in this work we mainly found a high level of caffeoyl derivatives [19]. 

*L. octovalvis* (*Jussiaea suffruticosa*) was reported by Murugesan et al. [20] as having antidiabetic activities in normal and alloxanized rats, but no action mechanism was proposed and no further research has been published to our knowledge. In the state of Morelos, it is employed as an infusion for dysuria due to prostate hyperplasia; this use is shared by some regions of the neighboring state of Guerrero. This plant contains C-glycosylated flavones like orientin, isoorientin, vitexin, and isovitexin, mainly [21]. According to our results, we found this kind of flavonoids in the hydroalcoholic extract, together with a high concentration of compounds with *λ* = 220, 275 nm. They could correspond to catechins [14].

Concerning the lipase inhibiting activity, three species yielded similar high values: *Artemisia absinthium, Acacia farnesiana *(L.) Willd, and* Tecoma stans* (25.2, 26.6, and 27.2% inhibition, resp.). Recently, Ikarashi et al. reported the presence of both inhibitory activities in the bark of *Acacia mearnsii* [22], identifying a catechin-rich preparation as the main active fraction. We found that leaves of *A. farnesiana* are also very active in lipase inhibition but low in glucosidase inhibition activities. In this case, the active extract mainly contains flavonol and flavone-like peaks (*λ* max = 210, 255, 355; 216, 271, 336 nm) [23]. *Tecoma stans* has been employed and studied as an antidiabetic for decades; we described the presence of glucosidase activity [12] and now we report the presence of a strong lipase inhibiting activity. Chromatographic analysis of this plant indicated a major concentration of caffeoyl derivatives and flavone-like compounds. 

The *in vitro* assay employed allowed us to identify highly active species in a fast, economical, and sensitive way. This approach does not substitute *in vivo* testing but acts as a prefilter of the chosen enzymatic activity and directs the animal models that, otherwise, may render negative results [24]. The lipase assay employed is sensitive and is not affected by zwitterionic compounds present in the extracts, but may be interfered by colored substances and free thiols, limiting its usefulness in some botanical species. 

In conclusion these results support the ethnomedical use of some plants reported by the Mexican traditional medicine and yield information about one of their action mechanisms that could be of immediate use to traditional healers. Although the major compounds in the most active species correspond to catechins, flavonols, flavones, and caffeoyl derivatives, these antidiabetic plants are being subjected to a detailed phytochemical and pharmacological study to identify their active compounds. 

## Figures and Tables

**Table 1:** Alpha-glucosidase and lipase inhibition of 23 medicinal plants used in Mexico for diabetes trearment.

Scientific name	Local name	Yield %	glucosidase % inhibition^a^	Lipase % inhibition^b^
*Acacia farnesiana *(L.) Willd.	Huizache	19.0	21.0 ± 3.1	26.6 ± 1.76
*Achillea millefolium *L.	Milenrama	27.3	52.3 ± 4.1	11.5 ± 2.67
*Annona squamosa* L.	Anona	16.4	22.3 ± 1.6	14.4 ± 3.0
*Artemisia absinthium *L.	Ajenjo	15.5	67.7 ± 3.7	25.2 ± 2.14
*Bidens pilosa *L.	Aceitilla	16.2	41.8 ± 1.7	13.6 ± 3.61
*Calea ternifolia *Kunth	Prodigiosa	18.4	61.1 ± 1.7	11.8 ± 0.18
*Camellia sinensis *(L.) Kuntze	Te Verde	35.5	85.0 ± 1.3	45.6 ± 4.31
*Cecropia obtusifolia *Bertol.	Guarumbo	18.6	28 ± 1.9	19.9 ± 5.4
*Hintonia latiflora *(Sessé & Moc. ex DC.) Bullock	Copalchi	41.3	39.2 ± 3.5	14.4 ± 2.8
*Crataegus mexicana *Moc. & Sessé ex DC.	Tejocote	27.5	38.6 ± 1.6	ppt
*Eucalyptus globulus *Labill*. *	Eucalipto	22.5	33.6 ± 3.0	11.3 ± 0.4
*Guazuma ulmifolia *Lam.	Guacima	15.4	23.0 ± 3.9	13.1 ± 1.37
*Iostephane heterophylla *(Cav.) Benth.	Zacapal	24.3	60.6 ± 1.5	15.1 ± 1.91
*Justicia spicigera *Schltdl.	Muicle	22.1	0.4 ± 1.5	12.7 ± 4.3
*Lepidium virginicum *L.	Lentejita	17.4	18.0 ± 1.1	9.6 ± 1.2
*Ludwigia octovalvis *(Jacq.) P.H.Raven	Clavillo	37.0	82.7 ± 1.9	31.4 ± 4.31
*Marrubium vulgare *L.	Marrubio	19.4	31.1 ± 2.3	1.8 ± 2.5
*Persea americana *Mill.	Aguacate	27.8	17.9 ± 2.9	6.3 ± 1.5
*Piper sanctum *Miq.	Hoja santa	22.2	10.5 ± 0.8	15.1±2.2
*Psidium guajava *L.	Guayaba	24.5	39.5 ± 3.0	ppt
*Ricinus communis *L.	Higuerilla	24.3	58.0 ± 1.9	14.4 ± 2.1
*Tamarindus indica *L.	Tamarindo	18.8	30.1 ± 2.2	ppt
*Taraxacum officinale *Webb	Diente de León	22.1	12.0 ± 1.5	5.0 ± 1.3
*Tecoma stans *(L.) Juss. ex Kunth	Tronadora	25.6	32.3 ± 1.7	27.2 ± 5.3

^a^ Percentage of *α*-glucosidase inhibition was calculated at *t* = 10 min, whereby the reaction = (mean free glucose in sample/mean free glucose in uninhibited control) × 100.

^b^ Percentage of lipase inhibition was calculated using the slope at *t* = 2–5 min, whereby the reaction = (mean slope in sample/mean slope in uninhibited control) × 100.

ppt: sample precipitation strongly interfered with lipase assay.

**Table 2:** *α*-glucosidase the 10 most active medicinal plants used in Mexico for diabetes treatment.

Scientific name	Local name	*α*-glucosidase % inhibition (0.5 mg mL^−1^)	IC_50_ (*μ*g mL^−1^)
*Artemisia absinthium*	Ajenjo	45.0 ± 1.7	n.d.
*Achillea millefolium*	Milenrama	22.9 ± 1.7	n.d.
*Calea ternifolia*	Prodigiosa	39.8 ± 2.1	n.d.
Camellia sinensis*	**Te Verde**	**67.3 **±** 0.8**	**299**
*Iostephane heterophylla*	Zacapal	51.4 ± 2.5	509
***Ludwigia octovalvis ***	**Clavillo**	**61.3 ±1.4**	**202**
*Ricinus communis*	Higuerilla	43.4 ± 2.4	n.d.

n.d.: no determined, *reference plant drug control. Percentage of *α*-glucosidase inhibition was calculated at *t* = 10 min as 100% reaction, whereby the reaction = (mean free glucose in sample/mean free glucose in control) × 100.
